# Headache impact and socioeconomic status: findings from a study of the German Migraine and Headache Society (DMKG)

**DOI:** 10.1186/s10194-023-01564-7

**Published:** 2023-04-04

**Authors:** Britta Müller, Charly Gaul, Olaf Reis, Tim P. Jürgens, Peter Kropp, Ruth Ruscheweyh, Andreas Straube, Elmar Brähler, Stefanie Förderreuther, Jennifer Schroth, Thomas Dresler

**Affiliations:** 1 Institute of Medical Psychology and Medical Sociology, University Medical Center Rostock, Gehlsheimer Str. 20, 18147 Rostock, Germany; 2 Headache Center Frankfurt, Frankfurt, Germany; 3 Department of Child and Adolescent Psychiatry and Neurology, University Medical Center Rostock, Rostock, Germany; 4 Department of Neurology, University Medical Center Rostock, Rostock, Germany; 5 Department of Neurology, University Hospital, Ludwig Maximilian University of Munich, Munich, Germany; 6 Integrated Research and Treatment Center (IFB) Adiposity Diseases - Behavioral Medicine, Psychosomatic Medicine and Psychotherapy, University of Leipzig Medical Center, Leipzig, Germany; 7 Department of Psychosomatic Medicine and Psychotherapy, University Medical Center of the Johannes Gutenberg-University, Mainz, Germany; 8 Clinic for Child and Adolescent Psychiatry, Psychotherapy and Psychosomatic Medicine, Brandenburg Medical School, University Hospital Ruppin-Brandenburg, Neuruppin, Germany; 9 Department of Psychiatry & Psychotherapy, Tübingen Center for Mental Health, University Hospital Tübingen, Tübingen, Germany; 10 LEAD Graduate School & Research Network, University of Tübingen, Tübingen, Germany

**Keywords:** Headache impact, Socioeconomic status, Obesity

## Abstract

**Backgound:**

Headache disorders are not only among the most prevalent, they are also among the most disabling disorders worldwide. This paper investigates the association between headache impact on daily life and the socioeconomic status (SES) of headache sufferers.

**Methods:**

Data stem from a random general population sample in Germany. Respondents who reported having headache for at least a year and were aged ≥ 18 years were included in the study. A standardized questionnaire addressing headache and headache treatment was filled in during the face-to-face survey. The impact of headache on daily life was measured using the German version of the Headache Impact Test (HIT-6).

**Results:**

Higher headache impact was found in low and medium SES compared to high SES. After adjustment for sociodemographics, headache-related factors (analgesic use, headache duration, headache frequency, migraine diagnosis), depressive symptoms, physical inactivity and obesity, an increased odds ratio of having higher headache impact in low SES compared to high SES was found: *OR* = 1.83, 95% *CI* [1.43, 2.23], *p* = .014. When the interactions "SES*obesity", "SES*depressive symptoms", and "SES*physical inactivity" were added, the results showed a significant interaction effect of “SES*obesity”. Obese persons with low SES were 3.64 times more likely to have higher headache impact than non-obese persons with low SES. No significant differences between obese and non-obese persons were found in the medium and high SES groups.

**Conclusions:**

SES is an important factor that should not be neglected in headache awareness campaigns and headache treatment. Longitudinal studies are needed in the future to investigate whether lifestyle interventions, such as weight reduction, can help to reduce headache impact in people in lower SES.

## Introduction

Worldwide, there is ample evidence for the association between socioeconomic status (SES) and health: the lower the individuals’ SES, the worse their health and the higher their mortality risk [1, 2, 3, 4]. Yet, in headache research, the question of whether headache impact is influenced by SES has hardly been studied. To the best of our knowledge, only one study has investigated the relationship between SES and headache impact [5]. We consider this fact regrettable, because headache disorders are not only among the most highly prevalent [6, 7, 8], but also among the most disabling disorders. Worldwide, an estimated 46.6 million years lived with disability (YLDs) is caused by headache disorders, 88% of which are due to migraine [9]. Headaches are the third leading cause of YLDs worldwide, after low back pain and depressive disorders [9]. Headache disorders impact personal life considerably, evident in absences from school, studies or work and reduced professional success [10, 11], limitations in social and leisure time activities [12], less family time [13], problems in partnership [14], and disability to do household chores [15]. Although pharmacological and non-pharmacological treatments can alleviate acute headache attacks or reduce the number of headache days [7, 16, 17], the substantial impact of headache disorders is still insufficiently alleviated by available therapies [18]. In many ways SES seems to be associated with headache impact, as either a result or a pre-condition. All kinds of impact listed here may be moderated or even mediated by SES. In our view, SES should be considered when identifying and treating individuals with high impact of headache.

**Fig. 1:**
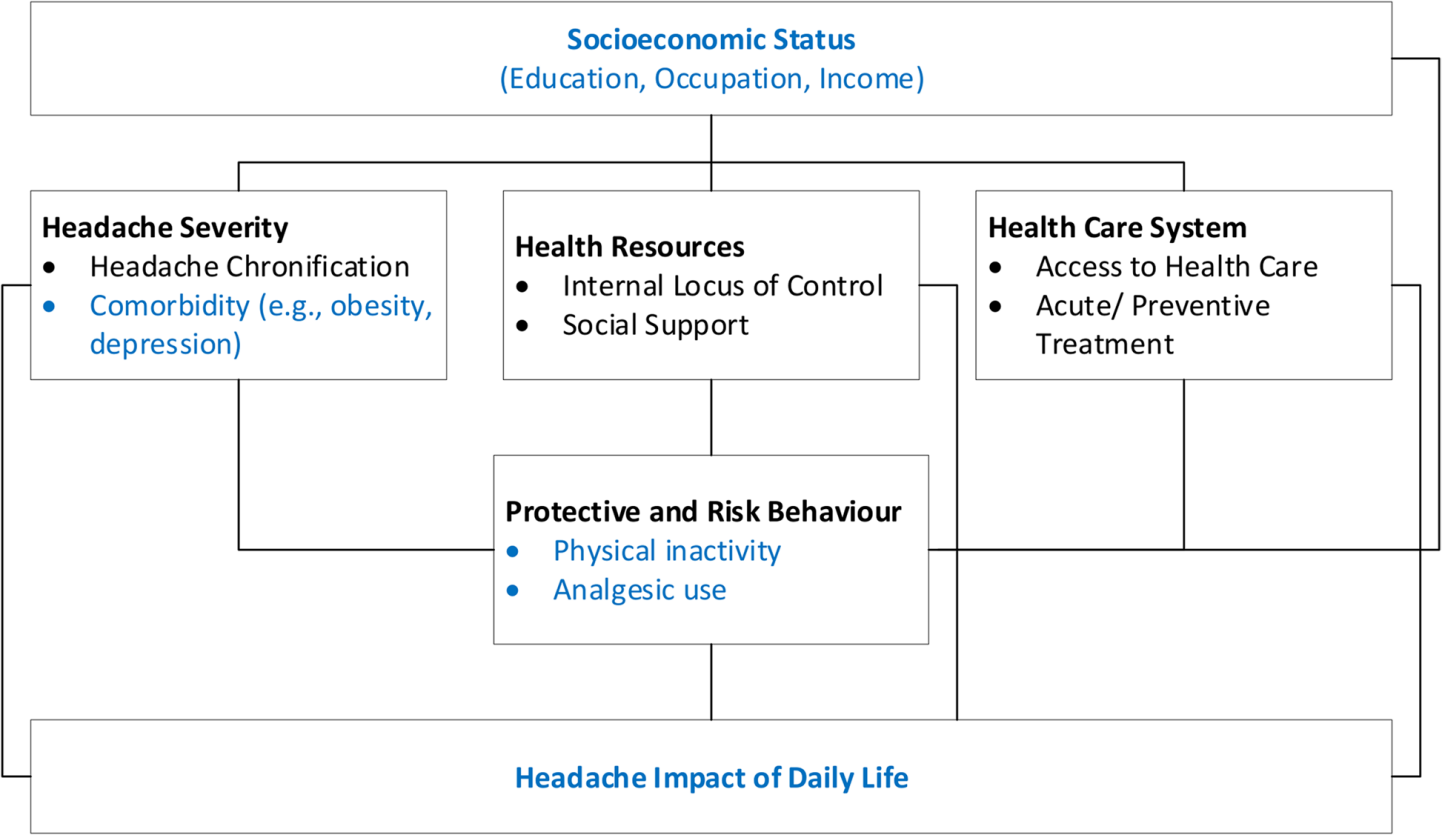
Potential mechanism linking SES and headache impact, based on the model of Elkeles and Mielck [19]

The primary objective of this study is to examine the relationship between headache impact and SES in a population-based study. The link between SES and headache impact is supported by several findings (see Fig. 1).

First, the severity of the headache seems to be important. The severity of the headache depends on the headache disorder. Migraine is more burdensome than tension-type headache (TTH) [20]. Indicators of high headache severity are chronic headache and comorbidities. Headache research has shown that depression [21, 22] and obesity [23] are common comorbidities of chronic headache. There is also evidence of an increased risk of chronic migraine in people with higher stress levels [24, 25, 26]. Chronic headache [27], obesity [28], depressive symptoms [5], and stress [29] are associated with higher headache impact. Studies outside of headache research have shown that people with lower social status both report more stressors and are more susceptible to them, leading to a higher risk of chronic stress [30, 31]. In addition, obesity [32] and depression [33] are more common in women with lower SES compared to women with higher SES.

Second, SES and headache impact could be linked through psychosocial health resources. Headache research suggests positive effects of internal Locus of Control (LOC) [34] and social support [35] on coping with headache disorders. More adaptive coping might result in lower level of headache impact. Research outside of headache research has shown that individuals in higher SES groups are more likely to have internal Locus of Control (LOC), more friends and more social support [36, 37].

Third, protective behaviour respectively risk behaviour may play a role in the association between SES and headache impact. Headache-related interventional studies suggest that physical activity may have beneficial influence on headache impact [38]. Furthermore, frequent use of analgesics may be significant. On the one hand, frequent use as a consequence of frequent headaches may reduce the headache impact. On the other hand, frequent use may lead to medication-overuse headache (MOH) [39, 40] and thus increase the headache impact [41]. Interestingly, low SES was identified as one of the risk factors for MOH [42]. Beside headache research, studies suggest lower levels of physical activity [43] and higher analgesic use [44] among those with lower SES compared to those with higher SES [43].

Fourth, a possible mechanism arises from characteristics of health care systems. Structural barriers to headache diagnosis and treatment may contribute to headache impact. Studies have shown that migraine patients with lack of insurance coverage are less likely to receive appropriate acute or preventive treatment in countries where income stewards the access to health resources [45, 46], and thus may be more affected by headache in their daily lives. Lueckmann, Hoebel [47] point out another aspect. In their systematic review, based on non-disease-specific articles, they found socioeconomic inequalities in the utilization of specialists: the highest SES groups were more likely to visit specialists.

Therefore, we expected SES and headache impact to be closely intertwined in a population-based sample of individuals suffering from headache. We assume that individuals with lower SES report greater headache impact compared to individuals with higher SES. Moreover, we investigated whether or not this association is linked to three factors that are strongly associated with headache impact: obesity, depressive symptoms and physical inactivity.

## Methods

### Participants

The analysis is based on cross-sectional data from a random general population sample (*N* = 2,510), collected in 2016 in Germany among inhabitants aged 14 years and older [48]. All participants gave their written informed consent. 900 participants reported having headache for at least a year. To be included in the analysis, respondents had to be at least 18 years of age and had to have provided complete information on headache impact and SES parameters (education, income, occupation). Out of the 900 respondents reporting headache for at least a year, 97 did not fulfil these criteria, resulting in 10.8% missing cases. The final sample consisted of 803 adult respondents.

### Questionnaire

A standardized questionnaire about headache and its treatment was used, which included sociodemographic variables [49]. The sociodemographic data were collected using face-to-face interviews. The questionnaire section on headache and its treatment was filled in by the respondents themselves.

#### Dependent variable

The impact of headache on daily life was measured using the validated German version of the Headache Impact Test (HIT-6) [50]. It consists of six items: pain, social functioning, role functioning, vitality, cognitive functioning and psychological distress. The total score ranges from 36–78. Higher scores indicate a greater impact of headache on the ability to function on the job, at school, at home and in social situations. The HIT-6 provides four grades indicating levels of headache impact: no or little impact (< 50), some impact [50–55], substantial impact [56–57] and severe impact (≥ 60).

#### Independent variables

Participants of the total sample were classified using the SES index of Lampert, Kroll [51]. This multidimensional index combines the three status dimensions “education”, “occupation”, and “income”. For each dimension, point values are assigned ranging from a minimum of 1 to a maximum of 7 (decimals allowed). In the present study, education and occupation were considered individual characteristics, whereas the dimension “income” was operationalized as a household characteristic. The scoring was done according to the procedure of Lampert, Kroll [51], which is based on international standards. For the dimension “education”, the Comparative Analyses of Social Mobility in Industrial Nations (CASMIN) was used [52]. The CASMIN classification distinguishes nine educational groups, taking into account both school education and vocational training. Information on school education was gained directly from participants. Vocational training (yes/no) was indirectly inferred from data on participants’ professional status. To classify professional status, standardized values were assigned to the surveyed occupational groups according to the Socio-Economic-Index of Occupational Index (ISEI) [53]. Data on household size and net household income were used to calculate the net equivalized income based on the OECD square root scale [54]. The general SES Index results from the addition of the three-point values and ranged from 3.0 to 21.0. Following the suggestion by Lampert et al. [51], we split the individuals into five equal groups (quintiles) according to their SES scores. Subsequently, the three middle groups (2nd to 4th quintile) were combined into one large “middle SES” group. As a result, a three-stage scale was obtained: low SES (first quintile; score 3.3–8.6, lower 20% of the population), middle SES (second to fourth quintile, score 8.7–13.6; middle 60% of the population), and high SES (fifth quintile, score 13.7–20.0, upper 20% of the population).

For headaches, four parameters were assessed. Duration was measured in years. Headache frequency was assessed using a five-point ordinal scale: (1) < 1 day a month; (2) 1–3 days per a month; (3) 4–14 days a month; (4) > 14 days a month but not daily; (5) and daily. For statistical analysis, the five categories were converged into three categories: < 4 days a month, 4–14 days a month, > 14 days a month. The kind of headache was assessed through diagnosis made by a physician as reported by the participants. The question used here was: “Do you know the diagnosis of your headache?” Response options were “migraine”, TTH, “cluster headache”, “other headache”, and “unclear headache (unknown diagnosis)”. As a fourth parameter on headache, acute treatment of headache was assessed with the question “How many days per month do you use analgesics on average?”.

Physical activity was surveyed with the dichotomous question: “Do you exercise regulary (i.e. on average at least 2–3 times a week for 30 min or longer)?” (Yes/ No). Participants were categorized as physically active if they answered “yes” and as physically inactive otherwise.

Self-report data on body weight and height were collected to calculate the Body Mass Index (BMI) (kg/m^2^). Obesity was defined as a BMI > 30 kg/m^2^ [55].

Depressive symptoms were measured with the subscale of the Patient Health Questionnaire (PHQ-4) that encompasses two items and has sum scores ranging from 0 to 6. Scores ≥ 3 indicate the presence of significant depressive symptoms. The scales showed acceptable reliability with McDonald's omega of ω = 0.85 for PHQ-4 [56]. Sociodemographic variables comprised sex, age, marital status, living with partner, minor children living in participant's household, school education, professional status, and net household income. The residential environment was classified into rural and urban areas based on the sampling plan. A rural region was defined as less than 20,000 inhabitants living in a community that was neither close to large cities nor part of a city-region or metropolitan area [58].

### Statistical analysis

The sample structure was compared with the population structure regarding a representative distribution by household size, age, sex, and federal states. To correct for deviations of the sample, a weighting factor was applied to improve the representativeness of the sample. All analyses were conducted with the weighting variable; absolute numbers of cases are presented unweighted.

To test for differences between SES groups, Pearson’s χ^2^ test and *F*-Test were used. The interpretation of results between categorical variables was based on the recommendations by Agresti and Kateri [59]. These authors suggest the use of adjusted standardized residuals (AR) to evaluate deviations between observed and expected frequencies. An adjusted residual exceeding 2 or 3 in absolute value indicates a rather unlikely deviation which can be interpreted as significant. In the present analysis, deviations exceeding a value of 2 were considered significant. Interpretations of results between metric and categorical variables was based on the Scheffé post-hoc method.

To test the hypotheses described above, ordinal logistic regression analyses were performed. The ordinal logistic regressions with headache impact as ordinal dependent variable (no or little impact: < 50, some impact: 50–55, substantial impact: 56–59, and severe impact: ≥ 60) and SES as independent variable (low, middle, and high SES) were sequentially adjusted for a set of sociodemographic variables (Model 2), headache-related variables (Model 3), other health-related variables (Model 4), and different single interaction terms (Model 5). Prerequisites of ordinal logistic regressions were tested. There were no violations of the assumption of no multicollinearity and proportional odds.

A *p* value < 0.05 was considered statistically significant. Statistical analyses were performed using IBM SPSS Statistics 27 (SPSS Inc., Chicago, IL, USA).

## Results

### Sociodemographic and health-related characteristics

The sociodemographic and health-related characteristics of the 803 participants according to SES are presented in Table 1. The following participants were over-represented in the low SES group: women, widowed and divorced participants, those living in a rural area, without partner, with headache frequency of > 14 and 4–14 days a month, physically inactive, and obese participants. Men, urban residents, participants with headache frequency < 4 days a month, physically active and non-depressed persons were more likely to be in the high SES group. Regarding age, post-hoc analysis revealed a significant difference between low and medium SES group (*p* = 0.003), and between low and high SES group (*p* < 0.001). Particpants in the low SES group were older than those in the medium and high SES groups. Mean level of analgesic use decreased from low to high SES group (*p* < 0.001), from low to medium SES group (*p* = 0.031), and from medium to high SES group (*p* = 0.031).

**Table 1:** Sociodemographic and health-related characteristics of the study sample according to the socioeconomic status (SES)

Variable	Total Sample	Socioeconomic Status (SES)			*p* value^a^
	*N* = 803	Low *n* = 156	Medium *n* = 482	High *n* = 165	
Sex (women), *n* (%)	535 (66.6)	117 (75.0)	321 (66.6)	97 (58.8)	.005^b^
(missing *n* = 0)					
Age, *M* (*SD*)	48.89 (15.6)	51.65(16.9)	48.93 (15.8)	46.15 (13.4)	< .001^c^
(missing *n* = 0)					
Marital status, *n* (%)					
Unmarried	217 (27.2)	42 (26.9)	122 (25.5)	53 (32.3)	< .001^b^
Married	394 (49.3)	57 (36.5)	256 (53.4)	81 (49.4)	
Divorced	123 (15.4)	37 (23.7)	60 (12.5)	26 (15.9)	
Widowed	65 (8.1)	20 (12.8)	41 (8.6)	4 (2.4)	
(missing *n* = 4)					
Living with partner (yes), *n* (%)	479 (59.9)	67 (43.2)	306 (63.7)	106 (64.6)	.001^b^
(missing *n* = 4)					
Children < 18 years (yes), *n* (%)	207 (25.8)	46 (29.5)	125 (25.9)	36 (21.8)	.571^b^
(missing *n* = 0)					
Living in an urban area (yes), *n* (%)	700 87.2)	126 (80.8)	422 (87.6)	152 (92.1)	.001^b^
(missing *n* = 0)					
Analgesic use (days a month), *M* (*SD*)	4.05 (5.9)	5.72 (8.0)	3.99 (5.5)	2.63 (4.1)	< .001^c^
(missing *n* = 15)					
Headache duration (in years)	13.13 (11.5)	13.46 (12.7)	12.60 (11.1)	14.39 (16.7)	.185^c^
(missing *n* = 0)					
Headache frequency (days a month), *n* (%)					
< 4	634 (79.7)	105 (67.7)	385 (81.1)	144 (87.3)	< .001^b^
4–14	129 (16.2)	33 (21.3)	77 (16.2)	19 (11.5)	
> 14	32 (4.0)	17 (11.0)	13 (2.7)	2 (1.2)	
(missing *n* = 8)					
Migraine (yes), *n* (%)	174(21.7)	41 (26.3)	101(21.0)	32 (19.4)	.335^b^
(missing *n* = 0)					
Physical activity (yes), *n* (%)	259 (32.4)	32 (20.6)	147 (30.6)	80 (48.8)	< .001^b^
(missing *n* = 3)					
Obesity (BMI ≥ 30) (yes), *n* (%)	158 (19.9)	47 (30.3)	82 (17.2)	29 (17.9)	.003^b^
(missing *n* = 9)					
Depressive symptoms (PHQ) (yes), *n* (%)	98 (12.3)	26 (16.8)	64 (13.3)	8 (4.9)	.003^b^
(missing *n* = 4)					

^a^Based on weighted sample

^b^Pearson's chi-squared test

^c^*F* test; *M*, Mean; *SD*, standard deviation

### Headache impact and SES

**Fig. 2:**
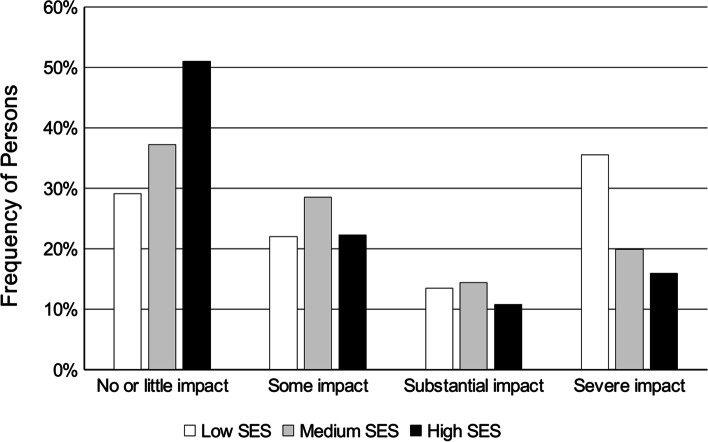
Proportions of individuals in headache impact categories depending on SES

38.5%, 26.0%, 13.5%, and 21.9% of the participants reported no/ little headache impact, some impact, substantial impact, and severe impact, respectively. There was a significant association between SES group and headache impact, χ^2^ (6) = 29.00, *p* < 0.001. Persons in the low SES group were over-represented among those with severe impact, persons in the high SES group were over-represented among those with no or little impact (see Fig. 2).

**Fig. 3:**
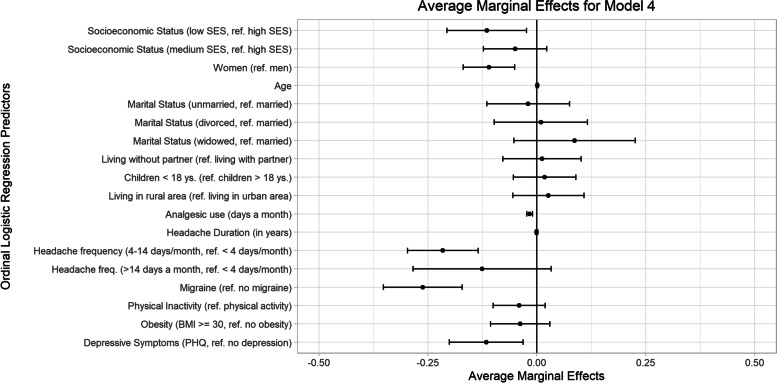
Average marginal effects (AMEs) of ordinal regression, model 4

Ordinal logistic regression models were conducted to investigate whether SES predicted the level of headache impact when additional factors are considered in a stepwise manner (Table 2). SES was found to be significantly associated with headache impact level. Compared to participants in the high SES group, participants in the low and medium SES groups were more likely to report higher levels of headache impact. (Model 1). This association remained statistically significant when sociodemographic variables were introduced to the equation (Model 2). When analgesic use, headache duration, headache frequency, and migraine diagnosis were added to the model, persons in the low SES group, but not in the medium SES group, were more likely to have higher headache impact level, compared to those with high SES (Model 3). This association was weakened but remained significant when physical inactivity, depressive symptoms and obesity were introduced to the prediction (see Model 4). A low SES was associated with an increased odds ratio of having higher headache impact: *OR* = 1.83, 95% *CI* [1.43, 2.23], *p* = 0.014. Other significant predictors were female sex, *p* < 0.001, more analgesic use, *p* < 0.001, higher headache frequency (4–14 days a month compared to < 4 days a month),* p* < 0.001, existence of a migraine diagnosis, *p* < 0.001 and being classified with depressive symptoms,* p* = 0.007. All coefficients can be found in Table 2. The average marginal effects of the full Model 4 are shown in Fig. 3.

**Table 2:** Ordinal logistic regression for the association between socioeconomic status (SES) and headache impact (HIT-6). Weighted random sample

	Model 1			Model 2			Model 3			Model 4		
	*b*	*SE*	95% *CI*	*b*	*SE*	95% *CI*	*b*	*SE*	95% *CI*	*b*	*SE*	95% *CI*
SES (ref. = high)												
Low SES	1.028***	.214	0.61, 1.45	.866***	.225	0.43, 1.31	.648*	.239	0.18, 1.12	.602*	.245	0.12, 1.08
Medium SES	.467**	.171	0.13, 0.80	.397*	.175	0.05, 0.74	.312	.183	-0.05, 0.67	.256	.189	-0.11, 0.63
**Other Sociodemographic variables**												
Sex (ref. = men)				.844***	.145	0.56, 1.13	.572***	.153	0.27, 0.87	.563***	.156	0.26, 0.87
Age				.002	.006	-0.01, 0.01	-.003	.006	-0.02, 0.01	-.005	.006	-0.02, 0.01
Marital status (ref. = married)												
Unmarried				.240	.236	-0.22, 0.70	.231	.252	-0.26, 0.72	.106	.258	-0.40, 0.61
Divorced				.100	.262	-0.41, 0.61	-.014	.275	-0.55, 0.53	-.049	.279	-0.60, 0.50
Widowed				-.093	.321	-0.72, 0.53	-.413	.358	-1.11, 0.29	-.448	.364	-1.16, 0.27
Living without partner (ref. = living with partner)				-.140	.216	-0.56, 0.28	-.150	.232	-0.60, 0.30	-.063	.237	-0.53, 0.40
Children < 18 years (ref. = no)				-.032	.175	-0.37, 0.31	.039	.186	-0.33, 0.40	.093	.191	-0.28, 0.47
Living in a rural area (ref. = living in an urban area)				.327	.196	-0.06, 0.71	.134	.212	-0.28, 0.55	.140	.214	-0.28, 0.56
**Headache-related variables**												
Analgesic use (days a month)							.096***	.015	0.07, 0.13	.086***	.016	0.06, 0.12
Headache duration (in years)							.003	.006	-0.01, 0.12	.002	.007	-0.01, 0.01
Headache frequency (ref. = less than 4 days a month)												
4–14 days a month							1.238***	.202	0.84, 1.63	1.215***	.210	0.80, 1.63
> 14 days a month							.706	.437	-0.15, 1.56	.650	.444	-0.22, 1.52
Migraine (ref. = no)							1.436***	.179	1.09, 1.79	1.495***	.178	1.15, 1.84
**Other health variables**												
Physical inactivity (ref. = no)										.212	.158	-0.10, 0.52
Obesity (BMI ≥ 30) (ref. = no obesity)										.200	.184	-0.16, 0.56
Depressive symptoms (PHQ) (ref. = no depressive symptoms*)*										.630**	.233	0.17, 1.09
Model fitting: χ^2^ (df)	22.70 (2) ***			58.67 (10) ***			259.58 (15) ***			272.34 (18) ***		
Goodness of fit												
(Pearson) χ^2^ (*df*)	3.85 (4)			1706.33 (1769)			2261.13 (2298)			2185.33 (2250)		
(Deviance) χ^2^ (*df*)	3.89 (4)			1551.05 (1769)			1710.96 (2298)			1653.16 (2250)		
Pseudo-*R*^2^ (Nagelkerke`s)	.031			.079			.316			.336		
Test of proportional odds: χ^2^ (*df*)	3.89 (4)			21.57 (20)			40.00 (30)			45.27 (36)		

*b* slope estimate, *SE* standard error, *df* degree of freedom; * *p* < .05; ** *p* < .01; *** *p* < .001; *CI* confidence interval, *ref.* reference, *BMI* body mass index, *PHQ* Patient Health Questionnaire, depressive subscale encompasses two items and has sum scores ranging from 0 to 6, scores ≥ 3 indicate depressive symptoms

### SES and interaction effects

**Fig. 4:**
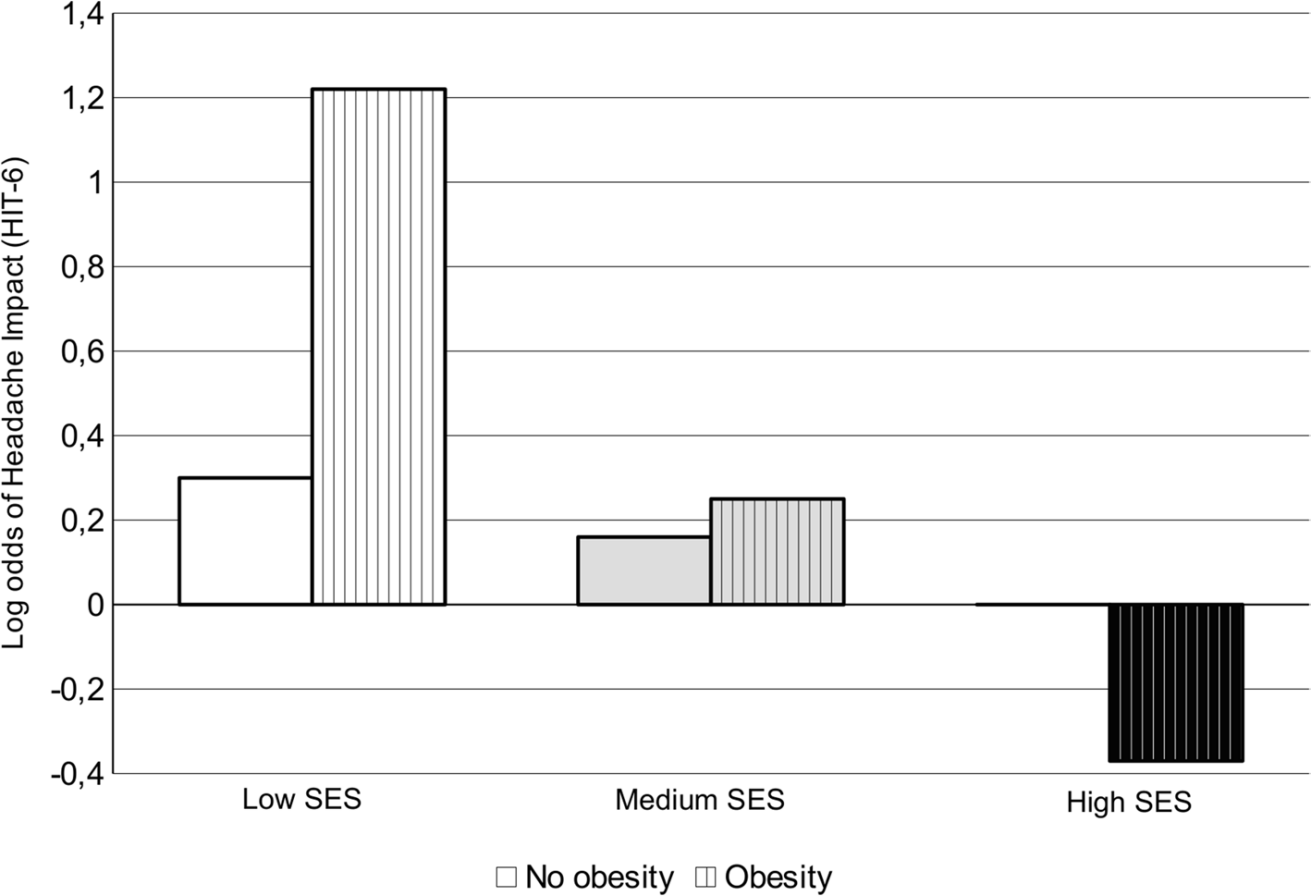
Predicted logits for each SES group and Obesity (yes/ no) combination

To test whether or not the association between SES and headache impact differs between participants with and without obesity, depressive symptoms and physical inactivity, one of the two-way-interactions between SES and obesity, SES and depressive symptoms, and SES and physical inactivity was added to each of the variables from Model 4 in three further ordinal logistic regressions (Model 5). The results indicate a significant interaction between SES and obesity on headache impact (see Table 3). There is no interaction effect between SES and physical inactivity and between SES and depressive symptoms (Tables 4 and 5 in the appendix). The significant interaction between SES and obesity relates to persons with low SES. Obese persons with low SES are 3.64 times more likely to have higher level of headache impact than non-obese persons in the low SES group, *OR* = 3.64, 95% *CI* [2.69, 4.59], *p* = 0.025. No significant differences between obese and non-obese persons were found in the medium and high SES groups, *OR* = 1.59, 95% *CI* [0.49, 2.39], *p* = 0.342, respectively *OR* = 0.69, 95% *CI* [0.04, 1.34], *p* = 0.393. When the interaction between SES and obesity was introduced into the equation, the single SES-factor was not found to be significant, *OR* = 1.34, 95% *CI* [0.88, 1.80], *p* = 0.286 (Tables 4 and 5 in the appendix). Figure 4 shows the relationship in terms of log odds.

**Table 3:** Ordinal logistic regression for association between socioeconomic status (SES) and headache impact (HIT-6). Addition of interaction “SES * obesity” to Model 4. Weighted random sample

	Model 5		
	*b*	*SE*	95% *CI*
SES (ref. = high)			
Low SES	.296	.277	-0.25, 0.84
Medium SES	.158	.209	-0.25, 0.57
**Other Sociodemographic variables**			
Sex (ref. = men)	.581***	.157	0.27, 0.89
Age	-.004	.007	-0.02, 0.01
Marital status (ref. = married)			
Unmarried	.125	.259	-0.38, 0.63
Divorced	-.038	.279	-0.59, 0.51
Widowed	-.491	.366	-1.21, 0.23
Living without partner (ref. = living with partner)	-.089	.238	-0.56, 0.38
Children < 18 years (ref. = no)	.079	.191	-0.30. 0.45
Living in a rural area (ref. = living in an urban area)	.153	.214	-0.27, 0.57
**Headache-related variables**			
Analgesic use (days a month)	.087***	.016	0.06, 0.12
Headache frequency (ref. = less than 4 days a month)			
4–14 days a month	1.222***	.210	0.81, 1.63
> 14 days a month	.485	.455	-0.41, 1.38
Migraine (ref. = no)	1.473***	.182	1.12, 1.83
**Other health variables**			
Physical inactivity (ref. = physical active persons)	.242	.159	-0.07, 0.55
Obesity (BMI ≥ 30) (ref. = no obesity)	-.368	.431	-1.21, 0.48
Depressive symptoms (PHQ) (ref. = no depressive symptoms*)*	.650**	.234	0.19, 1.11
**Interaction**			
SES * Obesity			
Low SES: obesity (ref. = no obesity)	1.292*	.577	0.16, 2.42
Medium SES: obesity (ref. = no obesity)	.462	.487	-0.49, 1.42
Model fitting: χ^2^ (df)	277.83 (20) ***		
Goodness of fit			
(Pearson) χ^2^ (*df*)	2202.79 (2248)		
(Deviance) χ^2^ (*df*)	1647.66.73 (2248)		
Pseudo-*R*^2^ (Nagelkerke`s)	.341		
Test of proportional odds: χ^2^ (*df*)	47.00(40)		

*b* slope estimate, *SE* standard error, *df* degree of freedom; * *p* < .05; ** *p* < .01; *** *p* < .001; *CI* confidence interval, *ref.* reference, *BMI* body mass index, *PHQ* Patient Health Questionnaire, depressive subscale encompasses two items and has sum scores ranging from 0 to 6, scores ≥ 3 indicate depressive symptoms

## Discussion

The study investigated the association between SES and headache impact – an aspect that has been largely neglected in headache research – and whether or not this association covaries with obesity, depressive symptoms and physical inactivity. Our results suggest that SES and headache impact are negatively correlated and that this relationship might be explained by obesity.

We provide evidence that individuals with low SES are more likely to have significantly higher headache impact than those with high SES. After controlling for sociodemographic, headache-related and health-related characteristics, this result remained significant but was smaller in size.

Consistent with our findings, Buse, Manack [5] reported higher headache impact in individuals with lower SES. Unlike their sample, our sample was not limited to persons with migraine. Futhermore, we controlled for additional variables, such as marital status, living with partner, younger children living in a participant's household, residential environment, analgesic use and physical activity. Our results suggest that the association between SES and headache impact is a fairly robust finding, which is not limited to migraine but seems to affect headache disorders in general. By providing evidence of interaction between SES and obesity on headache impact, we expand the results of Buse, Manack [5] and were able to identify one potential mechanism for the association between SES and headache impact.

Our study found that in the low SES group obese individuals had higher headache impact compared to non-obese individuals, an effect that was not found in the other SES groups. These results are similar to those reported by Kinge and Morris [57], who investigated the impact of obesity on health-related quality of life (HRQL) based on cross-sectional representative data of individuals living in England. In their non-disease-specific sample they found that obese individuals in lower SES group had lower HRQL than those of normal weight in the same SES group, and had lower HRQL compared to those in higher SES groups with the same weight. Subsequent studies confirmed these findings and found both moderator effects [60, 61] and mediator effects for obesity/ BMI [62] regarding the relationship SES and quality of life.

The main implication of our results is that SES is an important factor that should not be neglected in headache awareness campaigns and medical headache treatment. Our and other studies have provided evidence that particularly obese individuals with low SES suffer from severe headache-related impairments. Programmes in educational settings as introduced by the German Headache and Migraine Society (DMKG) could be one measure to motivate also persons with headache from the low SES group to contact their physician or to see a specialist concerning their headache. From a clinical point of view it is important that physicians should consider the socioeconomic context in which the patient lives. This could help them better understand the patient-defined needs and to make patient-centered treatment decisions. The SES can be identified through proxies such as occupation, insurance status, housing stability, and language [63]. It should be noted, however, that SES proxies might vary by country based on different welfare state contexts. Furthermore, when talking with low SES headache patients, physicians should adapt their communication style to the patient’s needs. In this way, a safe environment can be created that allows patients to talk about their concerns, needs and feelings. In addition, patients with low SES should be given more support to strengthen their resources in order to cope with headache [64].

Limitations of our study should be considered. First, we used cross-sectional data, which makes it difficult to draw conclusions about the causality of the relationship between SES and headache impact. A bidirectional relationship may be possible. Individuals with low SES are multi-deprived and suffer from more stressors (financial/ social problems) than individuals with high SES. Those with obesity have additional stressors (e.g., perceived stigmatization, additional obesity-related health problems). The consequences of headache in everyday life might be less compensable as a result of cumulative (disease and non-disease) mutually-reinforcing stressors [64]. On the other hand, a high headache impact could lead to unemployment or precarious employment, which would result in a socioeconomic decline with maladaptive coping behaviours (e.g., emotional eating) and a higher risk of obesity [65]. Furthermore, we consider it likely that the association between low SES and headache impact is mediated by several factors, such as depressive symptoms and reduced physical activity. Due to the cross-sectional research design of our study, we refrained from analysing mediator effects. However, it seems worthwhile to examine these effects in future longitudinal studies. Second, only self-reported data regarding headache-related variables (e.g., headache frequency, headache duration, migraine diagnosis) and health variables (physical activity, body weight and height) were analysed, which might be influenced by reporting bias. Third, with our analysis it is not possible to identify specific patterns of predictors for different headache disorders (e.g., migraine or TTH).

## Conclusion

Our study found that obese persons with low SES were more likely to have higher headache impact than non-obese persons with low SES. No significant differences between obese and non-obese persons were found in the medium and high SES groups. Based on our data, it would be desirable that prevention strategies to reduce the headache impact take into account the socioeconomic context of persons suffering from headache and pay particular attention to those with low SES.

Future studies based on longitudinal data should address whether lifestyle interventions, such as weight reduction, led to lower headache impact in people in lower SES. First data show that treatment of obesity either by conservative or surgical interventions does reduce headache frequency [28]. Headache impact is an aspect frequently analysed in headache research. Relationships between headache impact and SES have, however, received little attention. The present analysis suggests that it may be worthwhile to pay more attention to SES in headache research, especially in combination with obesity.

## Data Availability

The dataset generated and analyzed during this study is available from the corresponding author on reasonable request.

## Appendix

**Table 4:** Ordinal logistic regression for association between socioeconomic status (SES) and headache impact (HIT-6). Addition of interaction “SES * physical activity” to Model 4. Weighted random sample

	Model 5		
	*b*	*SE*	95% *CI*
SES (ref. = high)			
Low SES	1.158**	.436	0.30, 2.01
Medium SES	.382	.296	-0.20, 0.96
**Other Sociodemographic variables**			
Sex (ref. = men)	.578***	.157	0.27, 0.89
Age	-.005	.006	-0.02, 0.01
Marital status (ref. = married)			
Unmarried	.105	.260	-0.40, 0.61
Divorced	-.056	.279	-0.60, 0.49
Widowed	-.468	.365	-1.19, 0.25
Living without partner (ref. = living with partner)	-.053	.238	-0.52, 0.41
Children < 18 years (ref. = no)	.096	.191	-0.28, 0.47
Living in a rural area (ref. = living in an urban area)	.150	.214	-0.27, 0.57
**Headache-related variables**			
Analgesic use (days a month)	.086***	.016	0.05, 0.12
Headache duration (in years)	.001	.007	-0.01, 0.01
Headache frequency (ref. = less than 4 days a month)			
4–14 days a month	1.213***	.210	0.80, 1.62
> 14 days a month	.650	.443	-0.22, 1.52
Migraine (ref. = no)	1.479***	.182	1.12, 1.83
**Other health variables**			
Physical inactivity (ref. = physical active persons)	.484	.331	-0.17, 1.13
Obesity (BMI ≥ 30) (ref. = no obesity)	.184	.185	-0.18, 0.54
Depressive symptoms (PHQ) (ref. = no depressive symptoms*)*	.626**	.234	0.17, 1.08
**Interaction**			
SES * Physical inactivity			
Low SES: physical inactivity (ref. = physical active persons)	-.794	.521	-1.81, 0.23
Medium SES: physical inactivity (ref. = physical active persons)	-.241	.387	-1.00, 0.52
Model fitting: χ^2^ (df)	247.66 (20) ***		
Goodness of fit			
(Pearson) χ^2^ (*df*)	2187.42 (2248)		
(Deviance) χ^2^ (*df*)	1650.84 (2248)		
Pseudo-*R*^2^ (Nagelkerke`s)	.338		
Test of proportional odds: χ^2^ (*df*)	48.92 (40)		

*b* slope estimate, *SE* standard error, *df* degree of freedom; * *p* < .05; ** *p* < .01; *** *p* < .001; *CI* confidence interval, *ref.* reference, *BMI* body mass index, *PHQ* Patient Health Questionnaire, depressive subscale encompasses two items and has sum scores ranging from 0 to 6, scores ≥ 3 indicate depressive symptoms

**Table 5:** Ordinal logistic regression for association between socioeconomic status (SES) and headache impact (HIT-6). Addition of interaction “SES * depression” to Model 4

	Model 5		
	*b*	*SE*	95% *CI*
SES (ref. = high)			
Low SES	.618*	.254	0.12, 1.11
Medium SES	.233	.195	-0.15, 0.62
**Other Sociodemographic variables**			
Sex (ref. = men)	.559***	.156	0.25, 0.87
Age	-.005	.007	-0.02, 0.01
Marital status (ref. = married)			
Unmarried	.110	.259	-0.40, 0.62
Divorced	-.039	.279	-0.59, 0.51
Widowed	-.433	.365	-1.15, 0.28
Living without partner (ref. = living with partner)	-.058	.237	-0.52, 0.41
Children < 18 years (ref. = no)	.079	.191	-0.30, 0.45
Living in a rural area (ref. = living in an urban area)	.135	.214	-0.28, 0.55
**Headache-related variables**			
Analgesic use (days a month)	.086***	.016	0.05, 0.12
Headache duration (in years)	.002	.007	-0.01, 0.01
Headache frequency (ref. = less than 4 days a month)			
4–14 days a month	1.212***	.210	0.80, 1.62
> 14 days a month	.673	.448	-0.21, 1.55
Migraine (ref. = no)	1.485***	.182	1.13, 1.84
**Other health variables**			
Physical inactivity (ref. = physical active persons)	.213	.158	-0.10, 0.52
Obesity (BMI ≥ 30) (ref. = no obesity)	.203	.184	-0.16, 0.56
Depressive symptoms (PHQ) (ref. = no depressive symptoms*)*	.452	.752	-1.02, 1.93
**Interaction**			
SES * Depressive symptoms (ref. = high SES; no depressive symptoms)			
Low SES: depressive symptoms (ref. = no depressive symptoms)	-.077	.894	-1.83, 1.68
Medium SES: depressive symptoms (ref. = no depressive symptoms)	.273	.793	-1.28, 1.83
Model fitting: χ^2^ (df)	272.77 (20) ***		
Goodness of fit			
(Pearson) χ^2^ (*df*)	2183.62 (2248)		
(Deviance) χ^2^ (*df*)	1652.73 (2248)		
Pseudo-*R*^2^ (Nagelkerke`s)	.336		
Test of proportional odds: χ^2^ (*df*)	58.85 (40)*		

*b* slope estimate, *SE* standard error, *df* degree of freedom; * *p* < .05; ** *p* < .01; *** *p* < .001; *CI* confidence interval, *ref.* reference, *BMI* body mass index, *PHQ* Patient Health Questionnaire, depressive subscale encompasses two items and has sum scores ranging from 0 to 6, scores ≥ 3 indicate depressive symptoms

## Abbreviations

AR: Adjusted standardized residuals
BMI: Body Mass Index
CASMIN: Comparative Analyses of Social Mobility in Industrial Nations
HIT-6: Headache Impact Test
HRQL: Health-related quality of life
ISEI: Socio-Economic-Index of Occupatioonal Index
LOC: Locus of Control
MOH: Medication overuse headache
OR: Odds ratio
SES: Socioeconomic status
TTH: Tension-type headache
YLDs: Years lived with disability
